# Developing ultraviolet illumination of gillnets as a method to reduce sea turtle bycatch

**DOI:** 10.1098/rsbl.2013.0383

**Published:** 2013-10-23

**Authors:** John Wang, Joel Barkan, Shara Fisler, Carlos Godinez-Reyes, Yonat Swimmer

**Affiliations:** 1 Joint Institute for Marine and Atmospheric Research, University of Hawaii, Honolulu, HI 96822, USA; 2 Ocean Discovery Institute, San Diego, CA 92109, USA; 3 Comisión Nacional de Áreas Naturales Protegidas, Ensenada, Baja California, Mexico; 4 Pacific Islands Fisheries Science Center, NOAA Fisheries, Honolulu, HI 96822, USA

**Keywords:** sea turtles, bycatch, gillnets, UV vision

## Abstract

Fisheries bycatch of marine animals has been linked to population declines of multiple species, including many sea turtles. Altering the visual cues associated with fishing gear may reduce sea turtle bycatch. We examined the effectiveness of illuminating gillnets with ultraviolet (UV) light-emitting diodes for reducing green sea turtle (*Chelonia mydas*) interactions. We found that the mean sea turtle capture rate was reduced by 39.7% in UV-illuminated nets compared with nets without illumination. In collaboration with commercial fishermen, we tested UV net illumination in a bottom-set gillnet fishery in Baja California, Mexico. We did not find any difference in overall target fish catch rate or market value between net types. These findings suggest that UV net illumination may have applications in coastal and pelagic gillnet fisheries to reduce sea turtle bycatch.

## 1. Introduction

Incidental interactions between commercial fisheries and marine animals (e.g. seabirds, sea turtles and elasmobranchs) are linked to population declines in several vulnerable species [1–3]. In particular, several studies have shown that small-scale coastal gillnet fisheries may have high levels of sea turtle bycatch [3]. As such, fisheries bycatch is considered to be a barrier to the recovery of sea turtle populations [3] and has become a motivating factor to improve the balance between species protection and commercial fishing interests [4].

Coastal gillnet fisheries are ubiquitous [4]. Owing to the concern over high rates of sea turtle bycatch and mortality in several such fisheries [3], a variety of bycatch reduction technologies (BRTs), such as modifications to float lines [4], altering net tie-downs [4], use of at-sea advisory programmes [3] and net illumination [5] have been examined. One approach to developing BRTs for gillnet fisheries has been to better understand the sensory and behavioural ecology of sea turtles and target fish [6]. Identifying sensory cues that influences an animal's behaviour around fishing gear and understanding the animal's underlying sensory constraints can guide the development of BRTs [6]. Experiments have shown that changing the visual cues associated with fishing gear, such as illuminating nets with green light-emitting diodes (LEDs) or chemical lightsticks, can reduce green sea turtle (*Chelonia mydas*) interaction rates [5].

Anatomical, physiological and behavioural studies indicate that green, loggerhead (*Caretta caretta*) and leatherback (*Dermochelys coriacea*) sea turtles are sensitive to ultraviolet (UV) wavelengths [7]. While some fish species are also sensitive to UV light [8], several commercially valuable fish species are not [7,9,10]. Many of these fish species possess UV-absorbing compounds in their eyes that filter UV light and are thought to minimize damage from short-wavelength radiation [7–10]. Exploiting this disparity in visual capabilities between sea turtles that perceive UV light and fish without UV sensitivities may be a productive strategy in developing potential BRTs.

Our goal is to develop a BRT that reduces sea turtle bycatch without reducing the total target catch or the market value of catch. In this study, we examined the effects of illuminating gillnets with UV LEDs on sea turtle interaction rates. Separately, we also examined the effects of UV net illumination on target fish catch rates and catch value in a commercial gillnet fishery.

## 2. Material and methods

In 2011 and 2012, we conducted two experiments. One tested the effects of UV illumination on sea turtle capture rates in large mesh gillnets near Punta Abreojos, Baja California Sur, Mexico. The other experiment tested the impact of UV illumination on the total target fish catch and catch value in a commercial bottom-set gillnet fishery based in Bahía de los Angeles, Baja California, Mexico.

### (a) Testing net illumination effects on turtle catch rates

We deployed pairs of nets consisting of a control and an experimental net, to examine the effects of UV illumination on sea turtle catch rates. Experimental nets had UV LEDs (peak wavelength 396 nm) placed every 5 m on the floatlines. Control nets had inactive LEDs placed every 5 m. We used surface-set monofilament nets similar to those used to conduct green sea turtle population surveys [11]. The nets were 95 m long and 3 m deep with 40 cm mesh (stretched diagonal). After sunset, we deployed nets within approximately 1 km of each other and retrieved them before sunrise. We conducted experiments near Punta Abreojos, because the area has high densities of sea turtles [11], which ensures enough sea turtle interactions for robust analysis.

We checked the nets every 90 min. Sea turtles were removed from the nets, tagged, measured (straight carapace length) and released. We calculated sea turtle catch-per-unit-effort (CPUE) for each net as the number of turtles captured/([net length/100 m]) × ([net soak time/12 h]).

### (b) Testing net illumination effects on total target fish catch and catch value

We deployed pairs of control and experimental nets in a commercial bottom-set gillnet fishery. Experimental nets were illuminated with UV LEDs as described above, whereas inactive LEDs were similarly placed on control nets. Nets were 400 m in length, 1.5 m deep, with 1 m tie downs and with 8 cm mesh (stretched diagonal). We deployed nets 500 m apart in areas with similar depths and bottom habitat. Nets were deployed during the late evening, soaked overnight and retrieved at dawn.

We categorized all catch from the net into three groups: target species (fish sold), bycatch (discarded fish) and other (catch kept by the fishermen for consumption or retained for bait in other unrelated fisheries). We also followed the catch from each net to market in order to determine the catch value. Fishermen targeted species from a variety of taxa, primarily Pleuronectidae. We calculated the total target species CPUE as the number of individuals of target species/([net length/400 m] × [net soak time/12 h]). We calculated the value-per-unit-effort (VPUE) for each net as the market value (in US dollars) of the catch/([net length/400 m] × [net soak time/12 h]).

### (c) Analysis of sea turtle catch rates, total target catch and catch value

We used the randomization test to analyse the catch data and test the null hypothesis that there would be no difference in sea turtle catch rate, total target catch rate and VPUE of catch between experimental and control nets [12]. Data were resampled 10 000 times using the software Resampling Stats for Excel (v. 4.0). This analysis measures the strength of evidence against a null hypothesis instead of estimating significance at a certain level [13].

## 3. Results

**Figure 1.**
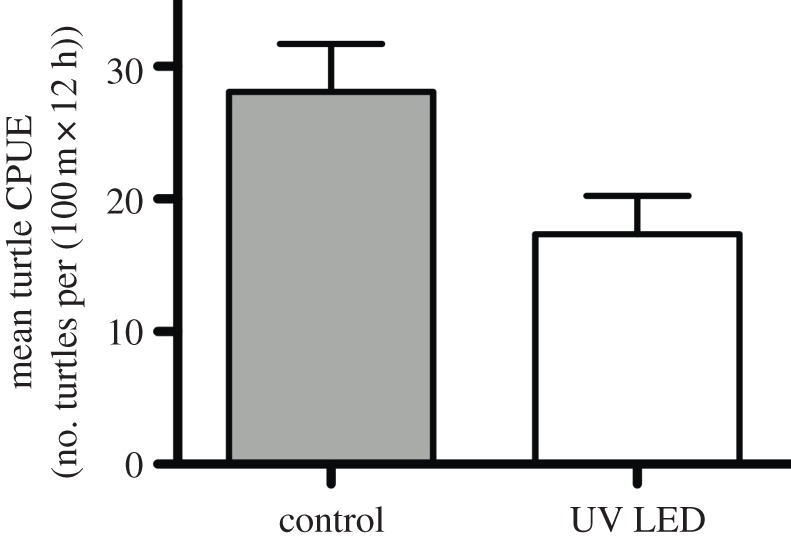
Effects of UV net illumination on sea turtle catch rates. Bars indicate s.e.

We deployed 11 net pairs, each consisting of a control and an experimental net, to examine the effects of UV net illumination on sea turtle catch rates. We caught 332 individual green turtles with 209 caught in the control nets and 123 caught in the experimental nets (see the electronic supplementary material, table S1). Sea turtle CPUE was significantly higher in control nets (mean CPUE = 26.7 ± 3.3 s.e.) as compared with experimental nets (mean CPUE = 16.1 ± 2.5 s.e.; *p* = 0.026), indicating a 39.7% reduction in mean catch rate (figure 1).

**Figure 2.**
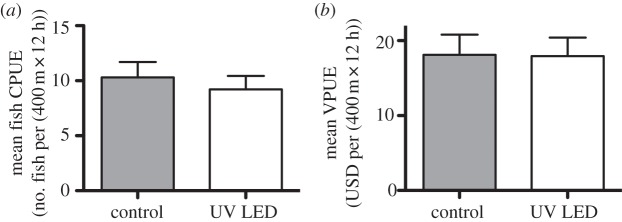
Effects of UV net illumination on (*a*) the total target catch rates and (*b*) catch value. Bars indicate s.e.

We deployed 36 pairs of nets to examine the effects of UV LED illumination on total target fish catch rates and catch value in a commercial bottom-set gillnet fishery. A total of 664 individual target fish were kept for market. Control nets caught 355 target fish (mean CPUE = 10.3 ± 1.4 s.e.), whereas experimental nets caught 309 target fish (mean CPUE = 9.22 ± 1.2 s.e.; figure 2*a*, see the electronic supplementary material, tables S1 and S2), which was statistically similar (*p* = 0.551). There was no significant difference (*p* = 0.420) in mean VPUE between the control ($15.1 ± 2.07 USD) and experimental nets ($15.0 ± 2.0 USD, figure 2*b*).

## 4. Discussion

In order to better balance species protection and commercial fishing interests, testing BRTs has become a priority in fisheries research [3,4,14]. Using visual cues to alert or deter bycatch species from fishing gear has been found to reduce bycatch in some fisheries [14–16]. Visual-based BRTs include the use of tori lines in longline fisheries [15] and the use of highly visible netting on gillnets to reduce sea bird bycatch [16]. Recent studies with nets illuminated with green LEDs or chemical lightsticks indicate that visual-based BRTs can also be effective for reducing sea turtle interactions [5]. Results from this study suggest that exploiting the differences in visual capabilities between sea turtles and fish species is a worthwhile approach for developing BRTs.

This study demonstrates that illuminating gillnets with UV light reduces sea turtle capture rates and, when tested in a commercial gillnet fishery, has no effect on total target catch rates and catch values in a commercial bottom-set gillnet fisheries. UV vision in sea turtles is thought to improve prey detection and potentially aid in navigation [3]. With regard to UV-illuminated nets, it is not known whether sea turtles avoid UV light or UV illumination merely helps alert sea turtles to the presence of the nets. Regardless, UV-illuminated nets may be a potentially useful BRT in coastal gillnets, though testing in gillnet fisheries with sea turtle bycatch issues must still be conducted.

The importance of engaging fishermen and their communities has been emphasized as an essential component to any BRT study [3]. Our study has included commercial gillnet fishermen as collaborators from its inception [5], and in doing so, these fishermen indicate that net illumination could be useful as long as net illumination does not adversely affect the overall value of their catch and adoption costs of the BRT are within reason. LED lightsticks are commonly used in commercial fisheries and costs have declined as LED technology matured.

Even though overall catch values were not affected with net illumination [5], fishermen were concerned that the catch of their primary target species may be negatively affected. Owing to differences in visual capabilities between fish species [8–10], it is reasonable to suspect that different wavelengths might induce different behaviours between teleost and elasmobranch species. While gillnets illuminated by green lights [5] and gillnets illuminated by UV LEDs reduce sea turtle interaction rates, they may affect different fish species in different ways. As such, it will be important to understand how the species composition of the target catch might be affected. In doing so, different wavelengths of light may be shown to improve the catch for particular species.

The efficacy of net illumination strategies may be influenced by factors associated within specific fisheries, such as environmental conditions (e.g. water transparency) and the visual capabilities of the local fish species. In addition, our experiments were conducted at night. While many gillnet fisheries occur primarily at night along the Baja coast, other gillnets fisheries are set over a 24 h period. The effectiveness of net illumination during daylight hours is unknown and requires further investigation.

A necessity for the development of BRTs is the need for high interaction rates with sea turtles in order for robust analyses. Even in fisheries with large numbers of estimated sea turtle interactions, catching an individual turtle is an infrequent event [12]. In order to develop and test potential BRTs, we used two different locations. One location had high densities of sea turtles to ensure sufficient catch rates necessary for robust comparisons and a second location that had a commercial gillnet fishery amenable to gear changes. Continued testing of UV net illumination will be required in actual fisheries with sea turtle bycatch concerns. As such, collaborations within those fishing communities must be established in a manner that balances sea turtle conservation goals while maintaining the viability of local fisheries and fishing communities.

## Notes

Procedures complied with UH-IACUC protocols.
